# Investigation into the role of the germline epigenome in the transmission of glucocorticoid-programmed effects across generations

**DOI:** 10.1186/s13059-018-1422-4

**Published:** 2018-04-10

**Authors:** Jessy Cartier, Thomas Smith, John P. Thomson, Catherine M. Rose, Batbayar Khulan, Andreas Heger, Richard R. Meehan, Amanda J. Drake

**Affiliations:** 1 University/British Heart Foundation Centre for Cardiovascular Science, University of Edinburgh, The Queen’s Medical Research Institute, 47 Little France Crescent, Edinburgh, EH16 4TJ UK; 2 MRC Computational Genomics Analysis and Training Programme, University of Oxford, MRC WIMM Centre for Computational Biology, The Weatherall Institute of Molecular Medicine, John Radcliffe Hospital, Headley Way, Oxford, OX3 9DS UK; 3 MRC Human Genetics Unit, Institute of Genetics and Molecular Medicine, University of Edinburgh, Crewe Road, Edinburgh, EH4 2XU UK

**Keywords:** Early life programming, DNA methylation, Histone modifications, Small RNA, Epigenetic, Germline transmission, Glucocorticoids

## Abstract

**Background:**

Early life exposure to adverse environments affects cardiovascular and metabolic systems in the offspring. These programmed effects are transmissible to a second generation through both male and female lines, suggesting germline transmission. We have previously shown that prenatal overexposure to the synthetic glucocorticoid dexamethasone (Dex) in rats reduces birth weight in the first generation (F1), a phenotype which is transmitted to a second generation (F2), particularly through the male line. We hypothesize that Dex exposure affects developing germ cells, resulting in transmissible alterations in DNA methylation, histone marks and/or small RNA in the male germline.

**Results:**

We profile epigenetic marks in sperm from F1 Sprague Dawley rats expressing a germ cell-specific GFP transgene following Dex or vehicle treatment of the mothers, using methylated DNA immunoprecipitation sequencing, small RNA sequencing and chromatin immunoprecipitation sequencing for H3K4me3, H3K4me1, H3K27me3 and H3K9me3. Although effects on birth weight are transmitted to the F2 generation through the male line, no differences in DNA methylation, histone modifications or small RNA were detected between germ cells and sperm from Dex-exposed animals and controls.

**Conclusions:**

Although the phenotype is transmitted to a second generation, we are unable to detect specific changes in DNA methylation, common histone modifications or small RNA profiles in sperm. Dex exposure is associated with more variable 5mC levels, particularly at non-promoter loci. Although this could be one mechanism contributing to the observed phenotype, other germline epigenetic modifications or non-epigenetic mechanisms may be responsible for the transmission of programmed effects across generations in this model.

**Electronic supplementary material:**

The online version of this article (10.1186/s13059-018-1422-4) contains supplementary material, which is available to authorized users.

## Background

Although development is a highly organised and tightly regulated process, the developing embryo is sensitive to environmental influences, resulting in pathophysiological changes which may increase the risk of later cardio-metabolic, neurobehavioural and reproductive disorders [1]. Effects on gene expression can persist after the removal of the inducing agent and be passed on through mitosis, and perhaps meiosis, to subsequent cell generations, which by definition represents a heritable epigenetic change [2]. Potential mechanisms have been proposed by which an initial environmental challenge may lead to epigenetic alterations which have direct effects on gene expression states in target tissues and might additionally directly influence cellular homeostasis in unexposed progeny [2]. For example, pharmaceutical-induced loss of promoter proximal DNA methylation relieves repression at a set of normally germline-specific genes in proliferating mouse embryonic fibroblasts [3]. Thus, environmentally induced changes in the epigenome may be an important indicator and mediator of such effects on the phenotype of exposed individuals and their progeny [4, 5].

A growing number of studies have shown that the effects of early life exposure to environmental influences are not limited to the first generation (F1), but may be transmitted to a second (F2) or further generations through non-genomic mechanisms [5–7]. Whilst transmission through the maternal line may be attributed to re-exposure via altered maternal physiology, or to changes in maternal behaviour [8, 9], paternal transmission in such animal models implicates effects transmissible through the germline, since in general in these models the male contributes little else to the offspring and its environment. Such data have led to the suggestion that induced epigenetic marks may be transmissible through the gametes [6, 10]. One possibility is presented by the enzyme-catalysed methylation of cytosines in DNA, which occurs at carbon 5 of the pyrimidine ring (5mC) through the actions of the DNA methyltransferase machinery. 5mC is a frequent and dynamic modification of DNA in many mammals, and is associated with transcriptional repression when present at regulatory regions. In the mouse, dynamic reprogramming of DNA methylation occurs following fertilisation and in the germline, and similar dynamic changes occur in human and rat development [11–13]. The erasure of DNA methylation and extensive chromatin remodelling that occur in primordial germ cells (PGC) is thought necessary to remove potential epimutations and to erase parental imprints [14]. Nevertheless, in PGCs, some regions escape this process, including potentially damaging retrotransposons and some loci associated with metabolic and neurological disorders [12, 15]. DNA de-methylation can occur passively through DNA replication or actively through oxidization of 5mC to 5-hydroxymethylcytosine (5hmC), 5-formylcytosine (5fC) or 5-carboxylcytosine (5caC) by the Ten-eleven translocation methylcytosine dioxygenases, Tet1–3 [16]. Aberrations at loci that are protected from this process could potentially be transmitted transgenerationally, and if associated with regulatory regions this may impact on expression states in cells carrying these abnormal epimodifications, as reported in reprogrammed cancer cells [17]. In plants, alternative modes of transgenerational transmission have been identified that are predicated on small inhibitory RNAs that can target the epigenetic machinery to unmodified loci in affected progeny [2], and recent data suggest that such mechanisms may also exist in mammals [18–20]. Finally, although most histones are replaced by protamines in sperm, some histones are retained at key loci and evidence suggests that alterations in sperm histones may underpin the transgenerational transmission of phenotypes [21–23].

Glucocorticoids play a key role during development to promote the maturation of organ systems, and exogenous glucocorticoid administration induces precocious maturation [24, 25]. However, prenatal glucocorticoid overexposure is associated with a reduction in birth weight in both animals and humans and has been associated with an increase in cardiovascular risk factors in adulthood [26, 27]. We have previously shown that this phenotype can be transmitted to a second (F2) but not a third generation: F2 offspring of male or female rats exposed to the synthetic glucocorticoid dexamethasone (Dex) also have a lower birth weight and exhibit hyperglycaemia in adulthood and the transmitted phenotype is stronger through the male line [26, 28]. Prenatal glucocorticoid exposure in rats also altered the expression and DNA methylation of candidate imprinted genes in the liver of F1 and F2 animals, suggesting an effect of prenatal Dex on the epigenome [28].

Our goal was to identify a potential mechanism for the transmission of the birth weight phenotype to a second generation through the male line. Since the germ cells which will form the F2 generation are also exposed to Dex, and this exposure occurs during the period when DNA methylation is re-established in the male germline [11], we hypothesised that prenatal glucocorticoid overexposure could disrupt i) DNA reprogramming in the male germline and/or alter ii) histone modification profiles or iii) small RNA (sRNA) expression in mature spermatozoa, facilitating the transmission of the programmed phenotype to a second generation. Our extensive analysis did not identify consistent differences between Dex-treated animals and associated controls. A major implication is that the inheritance mechanism for the paternally derived glucocorticoid-reprogrammed phenotype may not be linked with the specific germline DNA, sRNA and chromatin modifications that we have profiled here.

## Results

### Prenatal glucocorticoid treatment reduces birth weight in F1 and F2 generations

**Fig. 1:**
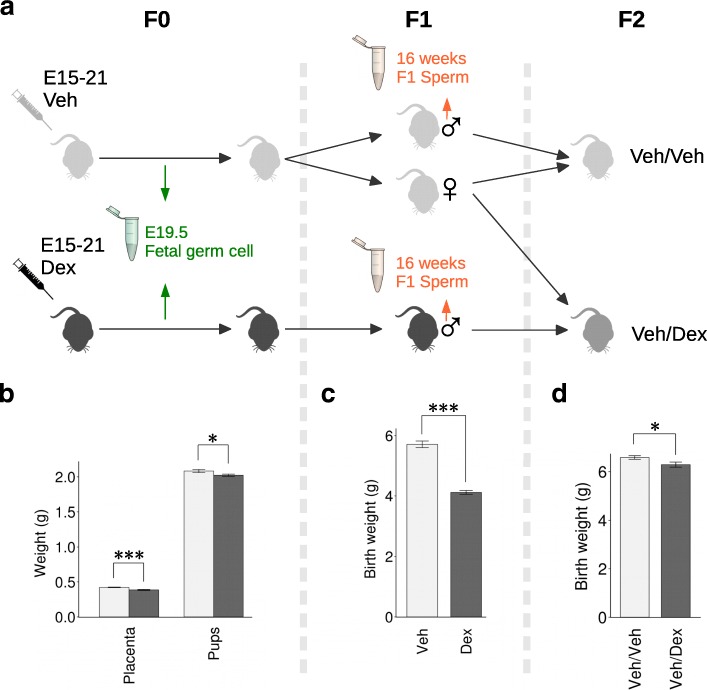
Experimental design and phenotype. **a** Experimental design. **b** Placenta and pup weight at e19.5 (*n* = 129 vehicle (Veh) and 120 Dex). Birth weight of **c** F1 (*n* = 75 Veh and 91 Dex offspring) and **d** F2 (*n* = 65 offspring from Veh mothers crossed with Veh fathers (Veh/Veh) and 77 offspring of Veh mothers crossed with Dex fathers (Veh/Dex)). Values represent mean weight ± standard error; *p < 0.05, ***p < 0.001 using unpaired Student *t*-test

The experimental design is summarised in Fig. 1a. Consistent with our previous studies [26, 28], pup and placenta weight was reduced at E19.5 in Dex-exposed pups (Fig. 1b) and birth weight was reduced in the F1 offspring of Dex-treated dams (Fig. 1c) and in the F2 offspring of F1 Dex males mated with F1 vehicle-treated (Veh) females (Fig. 1d).

### F1 sperm DNA methylation

**Fig. 2:**
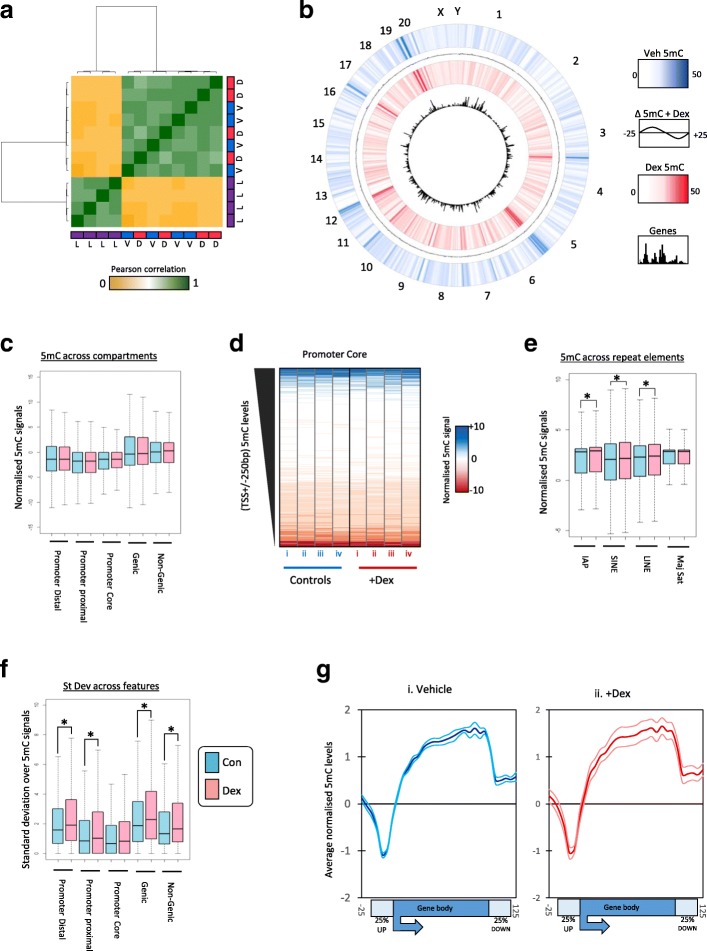
DNA methylation in F1 sperm is unaffected by Dex treatment. **a** Pearson correlation heatmaps with hierarchical clustering for 5mC datasets from sperm from offspring in which the mother had been exposed to dexamethasone (*D*) or vehicle controls (*V*) as well as in liver (*L*). **b** Circular visualisation of average meDIP datasets plotted as heatmaps. *Veh*, blue bars; *Dex*, red bars. Change in meDIP signal between Dex and Veh are plotted in *black* between the heatmap data. Positions of genes are shown in the inner circle. **c** Box plot of 5mC signals across one of five genomic compartments (“promoter core”, TSS ± 100 bp; “promoter proximal”, TSS + 1 kb; “promoter distal”, TSS + 1 kb to + 2 kb; “genic” or “non-genic”, not associated with any of the above). **d** Heatmap of average promoter core 5mC levels across sample sets. **e** Boxplot of 5mC signals across four common classes of repetitive element. **f** Boxplot of standard deviation scores between sample groups across genomic compartments. **g** Sliding window analysis of 5mC patterns (average patterns shown in *bold*, *upper* and *lower plots* denote upper and lower patterns using standard deviation scores between samples. In all plots *asterisks* denote *p* value < 0.05, Wilcoxon signed-rank test

To test if methylation patterns were altered in the sperm of F1 offspring, we carried out genome-wide methylated DNA immunoprecipitation followed by semiconductor sequencing (MeDIP-SC-seq) on four individuals per group [29]. As a comparison, we compared these patterns to those in a tissue with markedly different methylation patterns (liver) using recently published genome-wide datasets [30, 31]. Analysis of global methylation patterns through Pearson correlation analysis with Euclidian hierarchical clustering confirms that the sperm methylome differs dramatically from that of the liver (Fig. 2a). However, there was no clear stratification between the two groups of sperm samples (Fig. 2a). Differential signal analysis from average 5mC patterns between Veh- and Dex-exposed sperm revealed little difference in methylation across the entire genome (Fig. 2b and Additional file 1: Figure S1). In order to assess methylation patterns in more detail we mapped the data to one of five genomic compartments, three spanning promoter regions (“core”, transcription start site (TSS) ± 250 bp; “proximal”, 1 kb regions upstream of the core; “distal”, a further 1 kb upstream of proximal loci), one linked to coding “genic” loci and one linked to the remaining non-coding portions of the genome. Boxplot analysis of signals across these compartments highlights that in both sample sets methylation is lower over the promoter loci and enriched in genic and non-genic compartments (Fig. 2c). In agreement with the global analysis, across each compartment there was no significant difference in the levels of 5mC (*p* value > 0.05 Wilcoxon signed-rank test). Although we did not detect a strong change in signal across the compartments, a number of features could be changing in their absolute levels in opposite directions. As methylation at promoters has been functionally linked to changes in transcriptional states at associated genes, we focused on the 5mC levels across these loci in more detail. Heatmap visualisation of the 5mC signals reveals that aside from a small number of promoters, core signals are generally low in 5mC and do not display any clear changes in methylation levels upon Dex exposure (Fig. 2d). There was a small yet significant change in the levels of 5mC at a series of repetitive elements within the genome, particularly at intracisternal A particles (IAPs), small interspersed nuclear elements (SINE) and long interspersed nuclear elements (LINE) (*p* value < 0.05 Wilcoxon signed-rank test; Fig. 2e). Although we did not detect a clear change in methylation state across the genomic compartments, we did observe more variance in methylation levels in Dex-exposed littermates (significantly elevated standard deviation scores, *p* value < 0.05 Wilcoxon signed-rank test (Fig. 2f)), particularly across the bodies of genes (Fig. 2g). As such we deduce that a number of small but non-reproducible changes in 5mC levels occur following Dex exposure across the genome, particularly at non-promoter loci.

We have previously shown that global DNA methylation is re-established in the male rat germline during late gestation (embryonic day (E)15–E21) [11] and the prenatal glucocorticoid treatment applied here coincides with this period of germline methylome reprogramming. We therefore additionally sought to establish if glucocorticoid administration affected DNA remethylation in the germline even if the phenotypic effects in the F2 generation were not transmitted via DNA methylation changes. To test this we utilised enhanced reduced representation bisulphite sequencing (ERRBS) to interrogate CpG methylation for E19.5 fetal germ cells [32]. Again we observed no significant differences between Veh and Dex at the CpGs covered (Additional file 1: Supplementary methods and Figures S2 and S3).

### F1 sperm sRNAs

**Fig. 3:**
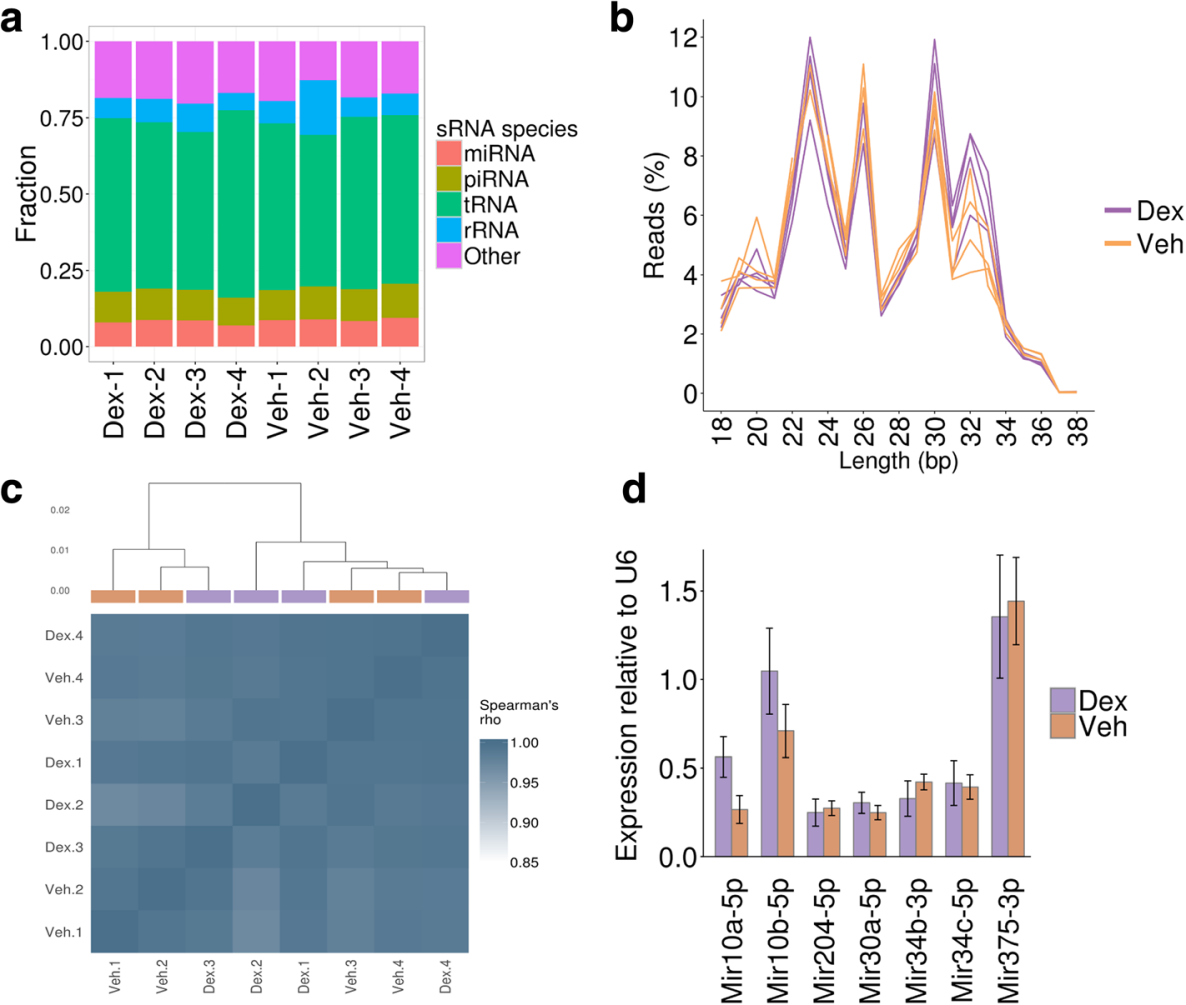
Small RNA expression in the F1 sperm is unaffected by Dex treatment. **a** The proportion of reads aligning to annotated small RNA species. Replicate samples are shown separately. **b** Length profile of sRNA-Seq reads following trimming to remove adapter read-through sequences. Reads exceeding 38 bp are not shown. Replicates are shown as separate lines. **c** Hierarchical clustering of Veh and Dex samples based on miRNA expression. Spearman’s correlation Rho shown below in heatmap. **d** Expression of candidate miRNAs in total RNA from sperm (*n* = 8/8). No significant differences were observed (Student’s *t*-test)

We next considered that Dex treatment might perturb the sperm sRNA profile, which could be responsible for the transmission of effects through the paternal line to the F2 generation. We utilised sRNA sequencing (sRNA-Seq) to quantify the expression of annotated sRNAs in F1 Dex and Veh sperm (four replicates each); in total 4.8–15.0 million mapped reads were obtained per sample. The sperm samples contained a very high proportion of reads aligning to tRNA-derived sRNAs (tsRNAs; derived from the 5′ half of tRNA sequences), in line with previous observations in studies of human and mouse sperm [33] (Fig. 3a). Between 5.6 and 9.4% of reads aligned to miRNA loci, 8.2–11.1% aligned to piRNA loci and 5.3–23.1% aligned to rRNA loci. Although the proportion of reads aligning to tRNA, miRNA and piRNA varied between samples, there were no consistent differences between the Dex and Veh replicates (Fig. 3a). Similarly, the length of the sRNAs sequenced was consistent between the Dex and Veh replicates (Fig. 3b). Taken together, these indicate that the prenatal Dex treatment does not induce a gross change in the sRNA profile in F1 sperm.

We counted reads aligned to annotated sRNA loci to identify differences in expression of sRNAs in Dex relative to Veh (see “Methods”). Hierarchical clustering of samples using the Spearman’s rank correlation between expression values did not separate the Dex and Veh replicates, suggesting the overall expression profile for these small non-coding RNAs in F1 sperm is not affected by Dex treatment (Fig. 3c). We used DESeq2 [34] to identify significantly differently expressed sRNAs between Dex and Veh with a false discovery rate of 10%. No sRNAs were identified as being significantly differently expressed, suggesting that Dex treatment did not specifically affect the expression of any particular sRNAs (Additional file 1: Figure S4a). We considered the lack of statistically significant differences identified may be due to a lack of power to detect changes in sRNA expression when controlling for multiple testing across the 25,642 annotated features included in our analysis. We therefore performed another simulation to estimate power (see “Methods”). We sampled from across the range of expression ranges in order to identify the level of fold change we were powered to detect and at what expression level. We estimate that we were 75% powered to detect a twofold change in expression for sRNAs with an average of 128 counts per sample, and greater than 50% powered to detect a fourfold change for sRNAs with an average of two counts per sample (Additional file 1: Figure S4b, c). This indicates that we were powered to detect the majority of changes in sRNA expression that could be expected to be biologically relevant.

Following personal communication with Oliver Rando (University of Massachusetts Medical School) we also repeated the entire analysis using an iterative mapping approach in which reads were mapped directly to the sequences of annotated sRNA loci (see “Methods”). Although the individual sRNA counts differed with the iterative mapping approach, the samples still did not cluster by treatment (Additional file 1: Figure S5) and no differentially expressed sRNAs were identified using DESeq2, again suggesting Dex treatment did not affect the expression of particular sRNAs in F1 sperm.

Finally, we performed RTqPCR for a number of candidate miRNAs chosen from the most expressed miRNA in spermatozoa, including Mir34c and Mir34b [35, 36], and found no differences between groups. Moreover, we confirmed the absence of changes in miRNAs affected by maternal (mir375) [37] or paternal stress (mir30a and mir204) [38]. Finally, there were no changes in expression of mir10a and mir10b, which are known to regulate hoxd10 [39], a gene belonging to the homeobox family, which is key to a number of developmental processes [40] (Fig. 3d).

### F1 sperm histone modifications

**Fig. 4:**
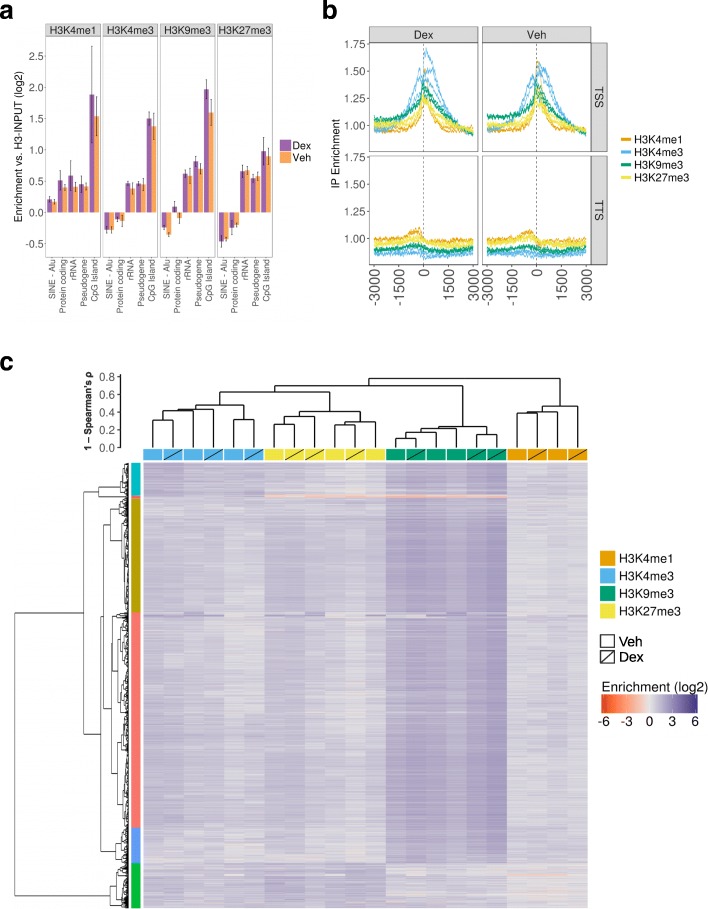
Dex treatment does not induce detectable changes in histone methylation. **a** Enrichment of methylated H3K IP over unmodified H3 IP for annotated features. Only features with at least 1.2-fold enrichment or depletion in one or more sample are shown. Error bars represent range for the three replicates. No significant differences were observed (Student’s t-test, Benjamini-Hochberg adjusted *p*-value, 10% FDR). **b** Enrichment of methylated H3K IP over unmodified H3 IP centred over transcription start sites (*TSS*) and transcription termination site (*TTS*) ± 3000 bp. Each replicate is shown as a separate line. **c** Hierarchical clustering of samples and peaks by average enrichment of methylated H3K IP over unmodified H3 IP. Samples clearly cluster by histone mark but do not cluster by Veh vs Dex for any histone mark. *Vertical colour bar* indicates six clusters following k-means clustering. *Gold* and *purple* clusters show higher H3K9me3 enrichment. Bivalent enrichment observed for H3K4me3 and H3K9me3 (*turquoise*) and H3K4me3 and H3K27me3 (*green*). *Blue* cluster represents inactive enhancers marked by H3K4me1 and H3K9me3

The vast majority of histones are replaced by protamines in mammalian sperm. Whilst the majority of histone retention occurs at large, gene-poor genomic regions [41], a small number of histones are retained at developmental promoters, where they may be important in the carriage of essential information to the early embryo [41–44]. We postulated that the reduced birth weight in the offspring of F2 offspring of F1 Dex males mated with F1 Veh females may be due to perturbed histone post-translational modifications in the F1 sperm. We performed ChIP-Seq for four histone modifications, H3K4me3 (active), H3K9me3 and H3K27me3 (both repressive) and H3K4me1 (which marks enhancers), in F1 Dex and Veh sperm, using unmodified H3 antibody as an input control. Three replicates were obtained for all histone marks, with the exception of H3K4me1 where two replicates were obtained. Following sequence quality filtering and alignment to the rn5 reference genome, we obtained 25.5–46.1 million mapped single end 50 bp reads (for complete alignment metrics see Additional file 2). We then computed the enrichment of the immunoprecipitation (IP) signal for the modified H3 marks relative to unmodified H3 over annotated features, including protein-coding genes, various repeats and retrotransposon classes and CpG islands (see “Methods”; Fig. 4a). A weak but consistent enrichment was observed for all three marks across CpG islands, rRNA genes and pseudogenes (Fig. 4a). A weak enrichment was also observed for H3K4me1 only over protein-coding genes and Alu elements. No significant differences were observed between the enrichment in the Dex and Veh samples at any annotated feature (one-way ANOVA with blocking, Benjamini-Hochberg adjusted *p* value). A weak enrichment was observed around the transcription start site (TSS), with H3K4me3 showing the expected dual peak in enrichment and H3K4me1, H3K9me3 and H3K27me3 enrichment centred on the TSS (Fig. 4b). None of the histone modifications were enriched at transcription termination sites. Again, there was no clear difference between the Dex and Veh samples.

In order to identify loci with high histone methylation, we identified enrichment peaks for each sample using SICER [45]. SICER was separately run in “Broad” and “Narrow” peak-calling modes to call between 32,058 and 100,290 peaks per sample. We then filtered the peaks into a low-confidence set (> 2-fold enrichment; 5146–48,739 peaks per sample) and high-confidence set (> 5-fold enrichment; 16–1585 peaks). Although H3K4me1 is an enhancer mark generally depleted at promoters, surprisingly in sperm, 4.1% of high-confidence H3K4me1 peaks were within ± 1000 bp of a TSS, significantly more than expected by chance (empirical *p* value from random sampling < 0.0001). We applied hierarchical clustering across samples and peaks using the estimated enrichments at all high-confidence peaks with at least ten reads in both inputs (2152 peaks in total); 1762 peaks (74%) show higher enrichment of H3K9me3 (Fig. 4c). The majority of remaining peaks appear to be bivalent, showing higher enrichment of H3K4me3 and H3K27me3 or H3K4me3 and H3K9me3 (10 and 7% of peaks, respectively). We also detected 172 peaks (8%) weakly enriched in H3K4me1 and strongly enriched in H3K9me3, which appear to represent inactive enhancers [46]. As expected, samples clearly clustered by the histone mark, but they did not further cluster by treatment (Fig. 4c), suggesting Dex treatment does not have a general affect on histone methylation.

We used MMDiff to identify specific loci with where Dex treatment altered the histone methylation profile using all peaks called in at least two samples. MMDiff inspects the difference between ChIP-Seq profiles and is designed to identify changes in either amplitude or shape of the peak profiles [47]. Applying MMDiff to the high-confidence peak set, we did not observe any significant differences between Dex and Veh for any histone modification. Extending the analysis to include low-confidence peaks did not yield any significant differences. Thus, Dex treatment was not observed to have any discernible general or loci-specific effect on histone modification in the F1 sperm.

Finally, we considered that changes in F1 sperm histone methylation may impact sRNA expression. Focusing on the 1000 bp immediately upstream of annotated miRNAs and piRNAs, we observed that fold changes in methylated histone IP enrichment between Veh and Dex did not correlate with expression fold changes between Veh and Dex (Additional file 1: Figure S6).

## Discussion

The aim of this study was to systematically profile the potential effects of *in utero* glucocorticoid exposure on the male germline to identify changes in DNA methylation, common histone modifications or sRNA, which may underpin the consistent transmission of glucocorticoid-induced effects on birth weight to a second generation through the male line [26, 28]. However, despite comprehensive profiling of a number of common modifications in sperm and germ cells, we were unable to detect any effect of glucocorticoid exposure in the male germline. Epigenetic inheritance is common in plants, where the germline arises from somatic cells late in development and can be influenced by the environment [2] and has also been demonstrated in *Caenorhabditis elegans* [48, 49], where the germline is set aside at the zygote stage and may be more easily influenced. In mammals, whilst germ cells transmit genetic information in the form of DNA from one generation to the next, the extensive reprogramming of the epigenome that occurs in PGCs and again following fertilisation, which is essential for erasing epigenetic memory, represents a major barrier to epigenetic inheritance. Nevertheless, some regions of the genome are known to resist this process [12, 15], and although it is unclear whether the environment can influence such regions, recent studies have suggested that acquired epigenetic marks can be transmitted across generations, influencing the phenotype of the offspring. However, there is ongoing robust debate over the importance of germline epigenetic effects in the non-genomic transmission of phenotypes across generations [50–53].

We have previously demonstrated altered gene expression and DNA methylation at candidate imprinted genes in F1 and F2 Dex-exposed offspring liver; notably, however, the direction of the changes in gene expression and the location of DNA methylation changes differed between the two generations and we were unable to detect specific methylation differences in sperm at the same loci [28]. In this study, expanding our search using both MeDIP-SC-seq and ERRBS has identified no sites of frequent DNA methylation change across the genome. Our results contrast with those reported in a model of maternal undernutrition in mice, which results in altered F1 male germline methylation at discrete loci, with locus-specific effects on gene expression in the F2 offspring that occur in the absence of persisting changes in DNA methylation [6, 54]. However, in this study the DNA methylation changes in the F1 sperm are low (10–30%) considering the penetrance of the phenotype, suggesting that DNA methylation may not be the epigenetic mark transmitting the phenotype to the F2 generation [6, 54]. In other models, exposure to excess glucocorticoids as a consequence of stress in mice has been shown to produce small changes in DNA methylation at candidate genes in the male germline and behavioural changes in offspring [55, 56] and in rats, exposure to the fungicide vinclozolin leads to effects on male fertility which persist for a number of generations in association with altered germline methylation [57]. However, in a recent detailed study using vinclozolin in mice, Iqbal and colleagues showed negligible effects on *de novo* DNA methylation and only subtle transcriptional changes in F1 prospermatogonia which were not seen in a second generation [58]. Further, despite the established precedent for transgenerational epigenetic inheritance at the *A*^*vy*^ locus in Agouti yellow mice, diet-induced *A*^*vy*^ hypermethylation is not transmitted across generations [59]. Such studies suggest that there are robust mechanisms in place to reset the germline epigenome and avoid the transmission of epigenetic changes to subsequent generations.

As an alternative mechanism to explain the transmission of effects we considered a role for sRNAs, which play a role in epigenetic inheritance in plants and in *C. elegans*, where piwi-interacting RNAs (piRNAs) can initiate highly stable, heritable epigenetic silencing in the germline which can persist for at least 20 generations [49]. Once established, this long-term memory becomes independent of the piRNA trigger but remains dependent on the nuclear RNAi/chromatin pathway [49]. A number of further studies suggest that sRNAs are responsible for the transmission of environmentally induced effects to progeny in this species [60, 61]; for example double-stranded RNA can be transferred from *C. elegans* neurons to the germline and cause transgenerational gene silencing [62]. In mammals, mature sperm also carries a significant population of sRNAs, including miRNA, piRNA and repeat associated sRNAs, which may be important in the post-fertilisation zygote. Exposure of pregnant female mice to vinclozolin leads to the specific dysregulation of miRNA in PGCs, with downstream effects on PGC differentiation, an effect which persisted for three generations [63]. In rodents, early life stress and dietary-induced obesity lead to altered expression of miRNAs in sperm, which may be responsible for the transmission of effects through the male germline [18, 37, 38, 64], and tsRNAs delivered into sperm by epididymosomes during maturation may additionally be important in the transmission of diet-induced effects [19, 20]. We were unable to identify changes in sRNAs in the germline despite performing deep sequencing and candidate gene analysis of miRNAs that are altered in other models.

Finally, we considered a role for altered histone modifications. In mammalian spermatogenesis, the majority of the histones are replaced by protamines to facilitate DNA compaction; however, some histones are retained, and disruption of histone methylation in developing sperm impacts on offspring health [21]. There are a few reports of alterations in sperm histones in animal models of induced phenotypic transmission, although the mechanisms by which they produce such specific effects in the offspring are unclear. For example, exposure to a high fat diet *in utero* is associated with altered histone H3 occupancy at key genes and with changes in H3K4me1 enrichment at transcription regulatory genes [22] and changes in histone modifications have been demonstrated at specific loci in rat sperm following cocaine administration [65] and induction of liver fibrosis [66]. Although we profiled a number of commonly studied histone modifications, including activating, repressive and enhancer-associated modifications, we identified no differences between Dex-exposed and control sperm.

Recent studies showing that “epivariation” between animals potentially exerts a stronger influence on the sperm epigenome than environmental exposures suggest that factors other than DNA methylation may account for the transmission of environmental effects on the phenotype to the offspring [67]. Although many groups have shown that the sperm methylome can be perturbed by environmental influences, including diet, stochastic epigenetic variation can affect the mouse sperm methylome to a greater extent than diet and this would be hard to reconcile with specific transgenerational outcomes that depend on fertilization by a single sperm [19, 67]. An alternative explanation is that transmission of the phenotype occurs in the absence of epigenetic perturbations in the exposed germline epigenome in this model. Alternative modes of transmission, as yet untested, include factors in seminal fluid, the influence of paternal behaviours on the mother, microbiome transfer or the transmission of metabolites [2, 68]. Nevertheless, it is possible that the transmission of Dex-induced effects on birth weight through the male germ line does indeed involve “epigenetic” mechanisms. We found a small change in the levels of 5mC at a number of repetitive elements and Dex exposure was associated with more variance in DNA methylation, particularly across gene bodies, suggesting that a number of small but non-reproducible changes in 5mC levels occur following Dex exposure across the genome. Although the meaning of these changes and any association with transmission of the programmed phenotype is unclear, it is possible that increased variation in 5mC at many disparate loci in Dex-exposed animals might impact on the expression of different weight-regulating genes and contribute to the F2 birth weight changes, even if there are no shared locus-specific changes. Additionally, we have not studied a number of other marks, including 5hmC, although this has been suggested as an unlikely mechanism for the germline transmission of effects since the levels of 5hmC are extremely low in the germline [6]. Further profiling of additional histone marks such as H3K27ac or protamine modifications may elucidate mechanisms for the transmission of effects in this model. Although the observed effect on birth weight is relatively small, we use this model because of its relevance to human populations, where the link between low birth weight and later cardiometabolic disease is seen for individuals with birth weights within the normal range. Differences in genetic background and treatment protocols have been suggested as explanations for the variability in findings in studies aimed at delineating epigenetic inheritance [53]; however, using this model we consistently see changes in birth weight transmitted across generations through the male line. Although it is possible that we failed to detect small epigenetic changes due to insufficient statistical power, we have demonstrated that we were sufficiently powered to detect changes in DNA methylation or in sRNA expression at levels that we would expect to be biologically relevant.

## Conclusions

Our data suggest that although glucocorticoid-induced effects on birth weight are transmissible to a second generation, this may not occur through changes in the germline epigenome and alternative mechanisms may explain the transmission of the phenotype through the male line in this model.

## Methods

### Ethics statement

All studies were conducted under licensed approval by the UK Home Office, under the Animals (Scientific Procedures) Act, 1986, and with University of Edinburgh ethical committee approval.

### Animals and treatment

Germ cell-specific eGFP (GCS-eGFP) rats [69], in which germ cells express eGFP (Fig. 1e, f), were maintained under conditions of controlled lighting (lights on 7:00 am to 7:00 pm) and temperature (22 °C) and allowed free access to food (standard rat chow, Special Diets Services, Witham, Essex, UK) and water. For breeding, a single virgin female was housed with a male in a breeding cage until an expelled vaginal plug was noted (designated embryonic (E) day 0); females were then housed singly until term (E21–22). Pregnant females (F0) were injected subcutaneously with dexamethasone (Dex) 100 μg/kg in 0.9% saline containing 4% ethanol (Dex mothers) or with an equivalent volume of vehicle (Veh; 0.9% saline containing 4% ethanol; Veh mothers) at the same time each morning between E15 and E21 inclusive. Females (*n* = 10 Veh and 9 Dex per group) were killed at E19.5; the pups and placenta were then weighed and sexed and males kept for testis extraction. A second cohort of pregnant females (*n* = 8 Veh and 8 Dex females per group) were allowed to deliver, and offspring (*n* = 75 Veh and 91 Dex) were weighed at birth and killed to leave 8/litter. For the second generation (F2), only the transmission through the male line was used. At maturity (90 days), F1 Veh females were timed-mated with F1 Veh or Dex non-sibling males giving F2 Veh (*n* = 6 Veh/Veh) and F2 Dex (*n* = 7 Veh/Dex). Females were caged separately during pregnancy and not manipulated in any way. We obtained a total of *n* = 65 F2 Veh/Veh and *n* = 77 F2 Veh/Dex offspring. Pups from F2 were weighed at birth.

### Sperm isolation

Sperm was isolated from the two epididymides of F1 Veh and Dex males at maturity (between 100 and 120 days). Each epididymis was sectioned and place in 10 ml of sperm swim buffer (DMEM F12 (Gibco, Life Technology, Paisley, UK), heat inactivated fetal calf serum (FCS; Hyclone) 5%, bovine serum albumin (BSA; Sigma-Aldrich, Dorset, UK) 2%) for 1 h at 37 °C with agitation at the start and end of the incubation. We transferred 8 ml of the upper supernatant into a clean tube and spun it for 5 min at 2000 g. The pellet was resuspended in 1 ml of somatic lysis buffer (0.1% SDS, 0.5% Triton X-100) for 5 min at room temperature. The sperm was then washed twice with 10 ml of phosphate buffer saline (PBS, Gibco) + 1% BSA (Sigma-Aldrich) and spun for 5 min at 2000×g. Sperm were counted using a hemocytometer and we obtained between 100 and 150 million sperm per animal. The purity of the sperm was assessed by FACScalibur (BD Biosciences, Oxford, UK).

### DNA isolation from sperm and meDIP

Genomic DNA was extracted from spermatozoa using the DNeasy Blood and Tissue Kit (Qiagen, Manchester, UK). Briefly, 10 M of spermatozoa in 100 μL were incubated with 100 μL buffer 2× (20 mM Tris HCl pH8, 20 mM EDTA, 200 mM NaCl, 4% SDS, 80 mM DTT, 12.5 μL/mL of Proteinase K (20 mg/mL; Qiagen, Manchester, UK)) at 56 °C for 1 h before adding 200 μL of AL buffer and 200 μL of 100% ethanol. From that point, the manufacturer’s instructions were followed. gDNA was fragmented using a COVARIS sonicator (Covaris Ltd, Woburn, MA, USA; peak incidence = 175, duty factor = 10%, cycles per burst = 205) and fragments from 150 to 400 bp were obtained prior to immunoprecipitation with anti-5mC (Eurogentec #BI-MECY-1000) antibody according to the procedure described [70]. Input and IP samples were amplified using a SEQXE WGA Kit (Sigma-Aldrich, Dorset, UK) before a clean-up step using a QIAquick Cleanup Kit (Qiagen, Manchester, UK). Samples were then sequenced on the Ion Torrent semiconductor sequencer using the Ion PI™ Hi-Q™ Sequencing Kit (Thermo Fisher Scientific, Paisley, UK) and an Ion PI™ Chip Kit v3 (Thermo Fisher Scientific, Paisley, UK).

### Small RNA isolation from sperm

Isolated sperm (100 million) were resuspended in 1 ml of Qiazol (Qiagen, Manchester, UK) with 100 mg of 0.2 mm stainless beads (Qiagen). The samples were then shaken for 2 min at 20 Hz using a Tissue Ruptor (Qiagen). The samples were kept for 5 min at room temperature after shaking, followed by the addition of 200 μl of chloroform. The samples were vortexed for 30 s and allowed to stand for 3 min at room temperature before being spun for 15 min at 16000×g. The aqueous superior phase containing the RNA was transferred to a new tube and sRNA isolated using the miRNeasy Mini Kit (Qiagen) according to the manufacturer’s instructions. sRNA quantity was assessed using a Qubit® 2.0 Fluorometer (Life Technology) and the quality assessed using the 2100 Bioanalyser (Agilent, Cheshire, UK).

### ChIP protocol

The protocol for ChIP on sperm was performed as described in Hisano et al. [71] with some modifications. Spermatozoa (100 million) were resuspended in 1 ml of 100 mM dithiothreitol (DTT; Sigma-Aldrich) in PBS and incubated for 2 h on a wheel at room temperature. DTT was quenched using 100 mM N-ethylmaleimide (NEM; Sigma-Aldrich) for 30 min at room temperature on the wheel. The spermatozoa were washed once with PBS, spun 5 min at 2000 g and resuspended in complete buffer 1 (15 mM Tris-HCl (pH 7.5), 60 mM KCl, 5 mM MgCl_2_, 0.1 mM EGTA, 0.3 M sucrose, 10 mM DTT) in a ratio of 100 μL/4 million cells. The cells were aliquotted in 100-μL aliquots with 100 μl of complete buffer 1 with detergent (15 mM Tris-HCl (pH 7.5), 60 mM KCl, 5 mM MgCl_2_, 0.1 mM EGTA, 0.3 M sucrose, 10 mM DTT, 0.5% (vol/vol) NP-40 and 1% (wt/vol) deoxycholate). Samples were vortexed well and incubated for 30 min on ice. After 30 min, 200 μl of MNase buffer (sucrose was added at a 0.3 M final concentration to the MNase buffer stock (85 mM Tris-HCl, pH 7.5, 3 mM MgCl_2_ and 2 mM CaCl_2_) and 60 units of MNase (Sigma-Aldrich) for every four million sperm to each of the tubes (200 μl/4 M cells) and vortexed. Tubes were placed at 37 °C for 5 min. The reaction was stopped by adding 4 μl of EDTA 0.5 M, vortexing and placing on ice for at least 5 min followed by centrifugation for 10 min at maximum speed at room temperature. The supernatants were then pooled. The chromatin was pre-cleared with 200 μl Protein A magnetic beads for 1 h at 4 °C on a wheel. Chromatin (1 ml) was dispensed into 1.5 ml tubes and 5 μg of each ChIP grade antibody added: H3K4me3 (Abcam, Cambridge, UK), H3K4me1, H3K27me3 (Millipore, Hertfordshire, UK), H3K9me3 (Abcam), H3 (Abcam) or Ig rabbit control (Abcam). Tubes were incubated overnight at 4 °C on a wheel. We retained 100 μl of the samples at this stage for use as the “input” sample for sequencing. The following day, the remaining samples were incubated for 2 h with 40 μl of protein A magnetic beads (Dynabeads, Life Technology) and then washed three times for 5 min each time on a wheel at 4 °C, once with buffer A (50 mM TRIS HCL pH 7.5, 10 mM EDTA and 75 mM NaCl+ Protease Inhibitor Complete (PIC, Roche)), followed by washing twice with buffer B (50 mM TRIS HCL pH 7.5, 10 mM EDTA and 125 mM NaCl + PIC). The beads were resuspended in 150 μl of elution buffer (100 μl of 10% SDS with 900 μl TE buffer) and incubated for 15 min on a wheel at room temperature. The supernatant was removed and kept and the elution was repeated a second time and the supernatants pooled. Input samples were made up to 300 μl with TE buffer. For all samples and input, 6 μl of RNAse A (10 mg/ml) was added and samples were incubated at 37 °C for 30 min, followed by the addition of 6 μl of proteinase K (Sigma-Aldrich). Samples were then incubated at 55 °C overnight. On the third day, ChIP DNA was purified using the PCR MinElute kit (Qiagen) according to the manufacturer’s instructions. The quantity of DNA was assessed using the Qubit® 2.0 Fluorometer (Life Technology) and the quality using the 2100 Bioanalyser (Agilent).

### Next-generation sequencing

MeDIP-SC-seq was carried out as described previously [29]. In brief 100 ng of DNA library for each sample was prepared using the Ion XpressPlus Fragment Library Kit (Thermo Fisher Scientific, Paisley, UK). The DNA was end repaired, purified and ligated to ion-compatible barcoded adapters (Ion Xpress™ Barcode Adapters 1–96; Thermo Fisher Scientific, Paisley, UK) followed by nick-repair to complete the linkage between adapters and DNA inserts. The adapter-ligated library was then amplified (ten cycles) and size-selected using two rounds of AMPure XP bead (Beckman Coulter) capture to size-select fragments approximately 100–250 bp in length. Samples were then pooled at a 1:1 ratio and sequenced on an Ion Proton P1 microwell chip (Thermo Fisher Scientific, Paisley, UK). Samples were sequenced to between 24 and 31 M reads. Sperm sRNAs and ChIP DNA were sent for next-generation sequencing at Source Biosciences (Nottingham, UK). Single-end 50-bp sequencing was performed on a HiSeq 2500 machine. We obtained 32.5–62.5 million ChIP-Seq reads and 8.6–20.9 million sRNA-Seq reads. All sequencing data can be accessed through the European Nucleotide Archive, accession number PRJEB14719 [72].

### Bioinformatics

Analyses of sRNA-Seq, ChIP-Seq and ERRBS data were performed with bespoke CGAT pipelines (https://github.com/TomSmithCGAT/Trans_of_gluco_effects_pipelines) utilising the CGAT code collection [73], CGAT pipelines repository (https://github.com/CGATOxford/CGATPipelines) and open-source software as detailed below. Analysis of MeDIP-Seq was performed using a previously reported approach [29] as detailed below.

### MeDIP-SC-seq analysis

Reads were mapped to the reference genome using the Torrent TMAP software. The data were then binned into 200-bp windows across the genome and data normalised first by read count and relative to a matched input sequence. These read count and input normalised datasets were then used for all subsequent analyses. Signals were then mapped to one of five unique genomic compartments (“promoter core”, TSS ± 100 bp; “promoter proximal”, TSS + 1 kb; “promoter distal”, TSS + 1 kb to + 2 kb; “genic” or “non-genic”, not associated with any of the above) using annotated Refgene_mm9 data supplied by the UCSC genome browser. Global MeDIP-SC-seq analysis was carried out by plotting Pearson correlation scores and representing these through heatmap visualisation with Euclidian clustering. Boxplots and heatmaps of 5mC levels (or standard deviation in 5mC signals) across genomic compartments were also carried out in R. Signals were also plotted over one of four classes of repetitive element using UCSC genome browser annotations. Average patterns of 5mC were plotted across length-normalised total gene sets (± 25% gene length) using the “sliding window over length normalised features” on our local GALAXY server, essentially plotting average patterns across these features. Average, upper and lower values per group were then plotted with respect to relative genomic location.

### Small RNA sequencing analysis

Quality of sequence reads was assessed with Fastqc v0.9.2. Reads were trimmed to remove adapters from read-through with trimgalore v0.32 with the following options: *ILLUMINACLIP:fasta.dir/contaminants.fasta:1:40:8 LEADING:3 TRAILING:3 SLIDINGWINDOW:4:15 MINLEN:18*. Reads were mapped to the rat rn5 genome using BWA [74] with the following options to set the seed length as 15, allow one mismatch in the seed and two mismatches in total: *aln -l 15 -k 1 -n 2 -t 12*. To assess the relative proportions of sRNA species per sample, reads with genomic alignments overlapping annotated sRNA loci were tallied. tRNA and rRNA annotations were obtained from the UCSC table browser. miRNA annotations for rn5 were obtained from Ensembl v78. Rn4 piRNA annotations were obtained from piRBase and converted to rn5 coordinates using CrossMap with the rn4 to rn5 liftover chain file from UCSC. sRNA expression was quantified using FeatureCounts v1.4.6 [75] with the following options to discard reads with a mapping quality < 10 and specify the sRNA-Seq strandedness: *-Q 10 -M -T 4 -s 1*. DESeq2 [34] was used to identify significantly differentially expressed sRNAs between the four Dex and Veh replicates. The DESeq2 rlog transformation was used to generate normalised counts, which were used for clustering and data exploration. Hierarchical clustering of samples based on expression of miRNA, piRNA or tRNA genes was performed using the R package pvclust, with 1000 bootstraps and the distance measure set as 1 − Spearman’s correlation coefficient.

To estimate our statistical power to detect differential expression we simulated *in silico* “spike-in” sRNA genes with differences in expression. To achieve this we shuffled the expression values between the sRNA genes for the Dex replicates whilst retaining the replicate structure to maintain the within-group variance. Spike-ins were binned by the mean expression and the induced fold change and randomly sampled to ensure even coverage over a range of expression values and fold changes. Spike-ins with fold changes greater than fourfold or expression greater than 1024 counts were discarded. Bins with fewer than 100 spike-ins were discarded and all remaining bins were downsampled to 100 spike-ins. In total, 2500 spike-ins were retained and added to the real sRNA expression data and the DESeq2 analysis repeated. Statistical power for a given bin was calculated as n/100, where n is the number of differentially expressed spike-ins detected.

Following personal communication with Oliver Rando (University of Massachusetts), we also quantified sRNA expression using an iterative approach to assign reads to sRNA. In addition, rather than quantifying against all annotated tRNA loci, we quantified tRNAs based on their 5′ 18-nucleotide sequence since the majority of reads aligning to tRNAs aligned to just the 5′ end of tRNA sequences, which is non-unique between tRNA loci. The maximum number of tRNA loci with an identical 18-nucleotide 5′ sequence is 28. Sequential rounds of mapping were performed. Reads were mapped first to rRNA sequences and unmapped reads were then mapped to tRNA sequences. Reads that remained unmapped were then mapped to miRNA sequences. This process was continued with piRNA sequences and finally a combined set of snRNA, scRNA, srpRNA and snoRNA sequences. This initial mapping was performed with Bowtie allowing one mismatch and retaining only reads mapping to a single sRNA sequence within a mapping round, e.g. “uniquely mapping”. Reads that did not map uniquely were sequentially remapped to the sequences in the same order but allowing reads to map to two sRNA sequences within a mapping round. In order to uniquely assign a read to a sRNA sequence, the read was randomly assigned to one of the two sequences with the probability of assignment derived from the number of reads which had previously been “uniquely” assigned to each of the sequences. This process was repeated with up to a maximum of 28 possible mapping locations with the probabilities for random assignment derived from the total number of previous assignments. The use of prior mapping information in an iterative approach has been previously implemented by the bowtie wrapper Butter [76]. However, our approach also enabled us to align to sRNA species in a sequential manner. The maximum depth of assigned reads across a sRNA sequence was taken as the expression estimate. Counts per tRNA loci sharing identical 18-nucleotide 5′ sequence were summed and these sequences became the unique tRNA identifiers. DESeq2 analysis and hierarchical clustering were performed exactly as described above for the sRNA quantification using BWA and featureCounts.

### Histone ChIP-Seq analysis

Quality of sequence reads was assessed with Fastqc v0.9.2. Reads were trimmed to remove adapters from read-through with trimmomatic v0.32 with the following options: *ILLUMINACLIP:contaminants.fasta:1:40:8 LEADING:3 TRAILING:3 SLIDINGWINDOW:4:15 MINLEN:30*. Reads were mapped to the rat rn5 genome using BWA [74] with the following options to set the seed length as 20 and allow two mismatches in the seed and five in total: *aln -l 20 -k 2 -n 5 -t 12.* We merged all Ensembl annotations (rn5, v78) with UCSC RNA and repeat annotations and computed the total read coverage for each feature by counting all reads which overlapped a feature for at least 50% of the read length. To compute the enrichment of IP over input we divided the IP counts by the count for their respective H3 input. To compute the meta-profile over gene models, we used the CGAT script bam2geneprofile.py which counts the reads overlapping the gene model, normalising each individual transcript profile by the maximum coverage and normalising the meta-profile to make the area underneath the curve equal to 1. To compute the enrichment of IP over input over the gene-model we divided the IP meta-profile by the meta-profile for their respective H3 input.

We utilised SICER [77] to call peaks in the histone modification samples relative to their respective H3 input sample, following the author’s recommendation to call peaks in both “narrow” and “broad” modes and keeping the peak calls from the two modes separate. Narrow peak calling was performed with the following options: *Redundancy_threshold = 1 Window size = 200 Fragment_size = 50 Gap_size = 200 False discovery rate controlling = 0.050000.* Broad peak calling was performed with the following options: *Redundancy_threshold = 1 Window size = 200 Fragment_size = 50 Gap_size = 600 False discovery rate controlling = 0.050000*. Low- (> 2-fold change) and high-confidence (> 5 fold change) peak sets were extracted by applying thresholds to the fold-change determined by SICER. Peaks were intersected with bedtools v22.0.

The enrichment of modified H3 IP over unmodified H3 IP for all high-confidence peaks observed in at least one sample was calculated for sample and peak clustering. Hierarchical clustering of samples and peaks was performed using the R function pv*clust*, using 1 − Spearman’s correlation coefficient as the distance and average linkage. The peak clusters were identified using the R function cutree (k = 5) and manually examined to determine their IP enrichment state. To test for significant overlap between H3K4me1 peaks and the TSS, we first identified the nearest TSS for each peak and classified peaks as TSS proximal (within ± 1000 bp) or distal. We then created 10,000 random sets of peaks with the same size and repeated the proximal/distal classification in order to obtain an empirical *p* value for the probability of obtaining the same or greater number of proximal peaks by chance.

We utilised MMDiff [47] to call significantly different histone methylation profiles between the Dex and Veh samples, performing the analysis separately for each histone mark, broad and narrow peaks, and low and high confidence peaks. For each MMDiff analysis, we included all peaks identified in at least two samples and set the false discovery rate threshold at 10% FDR.

## Additional files

Additional file 1: Supplementary methods. ERRBS on germ cells. **Figure S1.** 5mC profiling in sperm. **Figure S2.** DNA methylation in the developing germline is unaffected by Dex treatment across the genome. **Figure S3.** DNA methylation in the developing germline is unaffected by Dex treatment: reproducibility and power calculations. **Figure S4.** sRNA-Seq analysis is sufficiently powered to detect differential expression. **Figure S5.** F1-sperm sRNA expression shows consistent lack of affect for Dex treatment for two quantification methods. **Figure S6.** Fold changes between Dex and Veh sRNA expression and histone methylation and are not correlated. (PDF 1566 kb)

Additional file 2: Table of histone alignment metrics. (XLS 38 kb)
