# Supplementary material for: Investigation into the role of the germline epigenome in the transmission of glucocorticoid-programmed effects across generations
Source: Genome Biol. 2018 Apr 10;19:50. doi: 10.1186/s13059-018-1422-4 (PMC5891941; doi:10.1186/s13059-018-1422-4)

Cartier et al: Investigation into the role of the germline epigenome in the transmission of glucocorticoid-programmed effects across generations

A) Supplementary methods

ERRBS on germ cells

Germ cell isolation:

Testes from all males in a litter were collected and disrupted in 100 µl of collagenase IV (1 mg/ml in Hank's Balanced Salt Solution, Life Technology) followed by 5min at 37°C with regular shaking, to create a single cell suspension. eGFP-positive cells were then separated based on their emission wavelength (525±50nm for eGFP) using a FACSARIA II special order system (BD Biosciences). 4',6-diamidino-2-phenylindole (DAPI) was used as a marker of dead cells. Live, eGFP-positive singlets were gated into the sample, before centrifugation at 1200xg for 5 mins to yield a cell pellet. The identity of the cells was confirmed by qPCR for Dazl (Deleted in Azoospermia), a germ cell specific marker and Sox9 (Sry-Box9), a Sertoli cell-specific marker.

DNA isolation from germ cells:

DNA was isolated from germ cells using a protocol adapted from the Qiagen Gentra Puregene Handbook (2011) (Qiagen, Manchester UK). Germ cell aliquots were vortexed to loosen cell pellets before adding 300µl of Cell Lysis Solution (Qiagen, Manchester UK). Samples were vortexed for 10 seconds to promote cell lysis, before incubation at 37°C for 45mins followed by cooling on ice for 5mins. Following cell lysis, 0.75 µl of RNase A diluted to 4mg/ml in dH_2O was added to the sample which was then incubated at 37°C for 5mins. 100µl of Protein Precipitation Solution (Qiagen, Manchester UK) was added to the sample before vortexing vigorously for 20 seconds. Samples were centrifuged for 1 min at 16,000g and the supernatant placed in a new Eppendorf. 1µl of glycogen was added and DNA precipitated by the addition of 300µl of isopropanol, and mixing by inverting 50 times. DNA was pelleted by centrifugation for 10 min at 16,000xg, and washed by adding 300µl of 70% ethanol. Following further centrifugation, all ethanol was removed from the pellet and DNA was

dissolved in TE overnight at 4°C. DNA was quantified using a Qubit® 2.0 Fluorometer (Life Technology).

NGS sequencing:

Enhanced Reduced Representation Bisulphite Sequencing (ERRBS) was performed at Weill Cornell University Epigenetics Core, New York, USA. All sequencing data can be assessed through the European Nucleotide Archive, Accession PRJEB14719

ERRBS analysis

Analysis of ERRBS was performed with in-house pipelines utilising the CGAT code collection [69], CGAT pipelines repository (https://github.com/CGATOxford/CGATPipelines) and open-source software as detailed. Quality of sequence reads was assessed with Fastqc v0.9.2. Reads were trimmed to remove adapters and low quality bases using Trim-galore v0.3.3 with the following options: *-length 30 --rrbs -a AGATCGGAAGAGC --stringency 4*. Reads were mapped to the rat rn5 genome using the methylation-sensitive read mapper bismark v0.12.5 which maps reads to in-silico bisulphite converted genomes. The following options were used to align with bowtie2, set the seed length at 15 and allow a maximum of one mismatch in the seed alignment: *–bowtie2 -N 1 -L 15*. Between 59.6- 68.4% reads were aligned uniquely, to give 25.4 - 30.6 million aligned reads per sample. Reads containing 2 or more unconverted non-CpG cytosines were deemed to have originated from fragments which had failed to undergo full bisulphite conversion and were discarded. Methylation calls were made using bismark's bismark_methylation_extractor. It was noted that methylation values for the first 4 CpGs within a read were consistently much higher than the rest of the read. Thus, only CpGs from the 5th position onwards were retained by specifiying the option *- -ignore 4*. For all analyses, only CpGs with at least 10-fold coverage were retained. CpG clusters were defined as groups of >10 CpGs with less than 100bp between sequential CpGs. Hierarchical clustering was performed using the R function *hclust*, using Euclidean distance and average linkage.

High-CpG density promoters (HCP), Low-CpG density promoters (LCP) and Medium-CpG density promoters (MCP) were defined as described by Mikkelsen *et al* [70]. Differentially methylated regions (DMRs) were obtained from WAMIDEX atlas (https://atlas.genetics.kcl.ac.uk/ for the mouse genome (mm8) and sequentially lifted over to mm10 and finally rat (rn5). LTR, SINE, Alu elements, LINE and ERVK annotations were all obtained from UCSC. Highly Conserved Non-Coding Element (HNCE) annotations were obtained from CONDOR (http://condor.nimr.mrc.ac.uk/) [71].

Differential methylation analysis was performed with the Bioconductor package M3D (v1.3.4). M3D statistics were converted to empirical p-values by comparing M3D statistics from tests between pair of conditions to a null distribution of M3D statistics from test within conditions (between replicates) as described in Mayo et al 2015 [72]. P-values were adjusted for multiple testing using the Benjamini-Hochberg False Discovery rate (FDR) procedure and a FDR cut-off of 10% was applied to identify significantly differentially methylated clusters between pairs of conditions. The position of CpGs relative to annotated features was obtained using rn5 Ensembl v73 annotations. The simulation to assess statistical power was based on adding *in silico* "spike-in" clusters to the real eRRBS data. Spike-ins were generated by replacing the Dex replicate methylation values for 1-9 sequential CpGs from each cluster with methylation values from a random cluster. Spike-ins were then binned by the number of sequential CpGs shuffled and the magnitude of the methylation difference introduced. The procedure was repeated 10 times. The first 100 clusters per bin used in the power analysis. Where less than 100 clusters were generated for a given bin, the bin was not included in the power analysis. Statistical power for a given bin was estimated as n/100 where n is the number of differentially methylated clusters detected by M3D analysis.

ERRBS Results

We estimate that 4.6% of CpGs present in the rat genome are accessible to ERRBS, mainly at the GC rich regions associated with promoters and enhancer regulatory regions, which we would predict to be potential target regions for transmissible epigenotypes. We compared the DNA methylation profiles between Dex and Veh samples at increasing levels of detail. Promoter methylation was anti-correlated

with CpG density, with methylation highest at low-CpG density promoters and higher over low-CpG density LTRs, LINEs, SINEs and Alu elements (Additional file 1: Figs. S2A and S2B). High CpG density promoters were almost entirely unmethylated and there was an anti-correlation between repeat and retrotransposon methylation and CpG density (Additional file 1: Fig. S2B). Methylation distributions were very similar between germline Veh and Dex, indicating that prenatal Dex treatment does not have a uniform impact on germline re-methylation (Additional file 1: Fig. S2C). To identify regions of the genome which may be differentially methylated following prenatal Dex treatment, CpGs were grouped into clusters based on the following thresholds: 10 or more sequential CpGs, each separated by less than 100 bp, each with greater than 10-fold coverage. On hierarchical clustering using Euclidean distance and average linkage for mean methylation values at each cluster, replicate samples showed a good correlation between single CpG resolution methylation estimates however Veh and Dex replicates did not separate, indicating that the prenatal Dex treatment did not cause widespread changes in DNA methylation in the germline (Additional file 1: Fig. S2C). Correlations between replicates were high for CpG island CpGs and all other CpGs (Additional file 1: Fig. S3A). To investigate whether more local changes could be identified, we used M3D to compare DNA methylation profiles over CpG clusters [30]. This approach is preferred over comparing methylation at the per-CpG level since it significantly reduces the burden of multiple testing and DNA methylation is correlated over short distances [31]. Again, no significant differences were observed between the Veh and Dex treatments in the germline. These results suggest that prenatal Dex treatment does not disrupt germline DNA re-methylation at the CpGs interrogated.

To establish that we had not missed local changes within CpGs using a genome-wide statistical approach, we also tested for significant changes in promoter or gene-body DNA methylation at two genes known to be imprinted (Igf2r and Smfbt2) for which we had coverage over the previously defined DMRs, and established there was no change in DNA methylation between Veh and Dex in the germline (Additional file 1: Fig. S3B). To confirm that we were powered to detect biologically relevant changes in DNA methylation in the germ cell samples, we performed a simulation study to estimate power by shuffling DNA methylation values but maintaining the replicate structure, thus

introducing artificial fold-change differences but retaining the variation in expression between replicates (see methods above). As expected, we had better power to detect larger changes in DNA methylation (Additional file 1: Fig. S3C). 80% power was reached where the average change in methylation over 6 CpGs was 25%, or 35% over 3 CpGs. Thus, we were suitably powered to detect the majority of any biologically relevant changes in DNA methylation that may have occurred within CpG clusters as a result of prenatal Dex exposure.

B) Supplementary Figures

Figure S1: (A) Visual example of 5mC levels across the highly methylated imprinted gene Igf2 in Veh (blue) and Dex exposed (red) sperm datasets. Differential methylation plots between average sample sets are shown in black. Input normalised reads range from -50 to +50. (B) Scatter plots with r and cor values for normalised 5mC windows in all samples

A

B

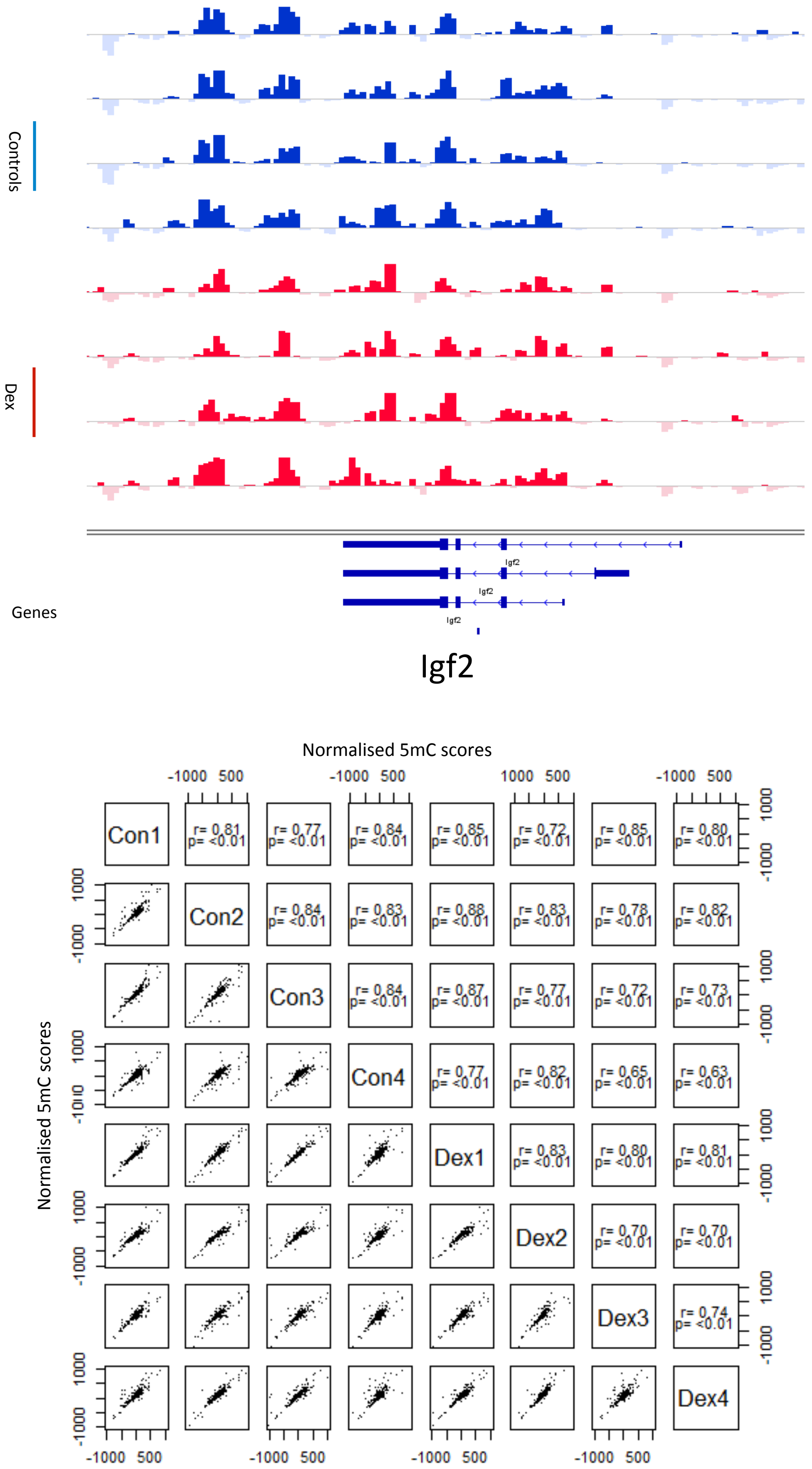
Controls
Dex
Genes
Igf2
Normalised 5mC scores
Normalised 5mC scores
-1000 500
1000
-1000
Con1
Con2
Con3
Con4
Dex1
Dex2
Dex3
Dex4
r= 0.81 p= <0.01
r= 0.77 p= <0.01
r= 0.84 p= <0.01
r= 0.85 p= <0.01
r= 0.72 p= <0.01
r= 0.85 p= <0.01
r= 0.80 p= <0.01
r= 0.84 p= <0.01
r= 0.83 p= <0.01
r= 0.88 p= <0.01
r= 0.83 p= <0.01
r= 0.78 p= <0.01
r= 0.82 p= <0.01
r= 0.84 p= <0.01
r= 0.87 p= <0.01
r= 0.77 p= <0.01
r= 0.72 p= <0.01
r= 0.73 p= <0.01
r= 0.77 p= <0.01
r= 0.82 p= <0.01
r= 0.65 p= <0.01
r= 0.63 p= <0.01
r= 0.83 p= <0.01
r= 0.80 p= <0.01
r= 0.81 p= <0.01
r= 0.70 p= <0.01
r= 0.70 p= <0.01
r= 0.74 p= <0.01

Figure S2: DNA methylation in the developing germline is unaffected by Dex treatment. (A) Distribution of percentage methylation within each sample. Left panels show CpG islands, right show non-CpG islands. (B) Top panel shows the distribution of methylation (%) across HCPs, LCPs, HCNEs, differentially methylated regions (DMRs), LTRs and SINE, Alu and LINE elements. n = number of CpGs. Bottom panel shows the distribution of methylation (%) conditional on local CpG density (see methods). Boxes denote the interquartile ranges. Whiskers extend to 1.5 * Inter-Quartile Range (IQR) from the box hinge. Red lines denote median, notches are rough 95% confidence limits (median ± 1.58* IQR). (C) Hierarchical clustering of samples based on mean methylation values over CpG clusters. Colour bar represents tissue and condition. Columns = samples, rows = CpG clusters.

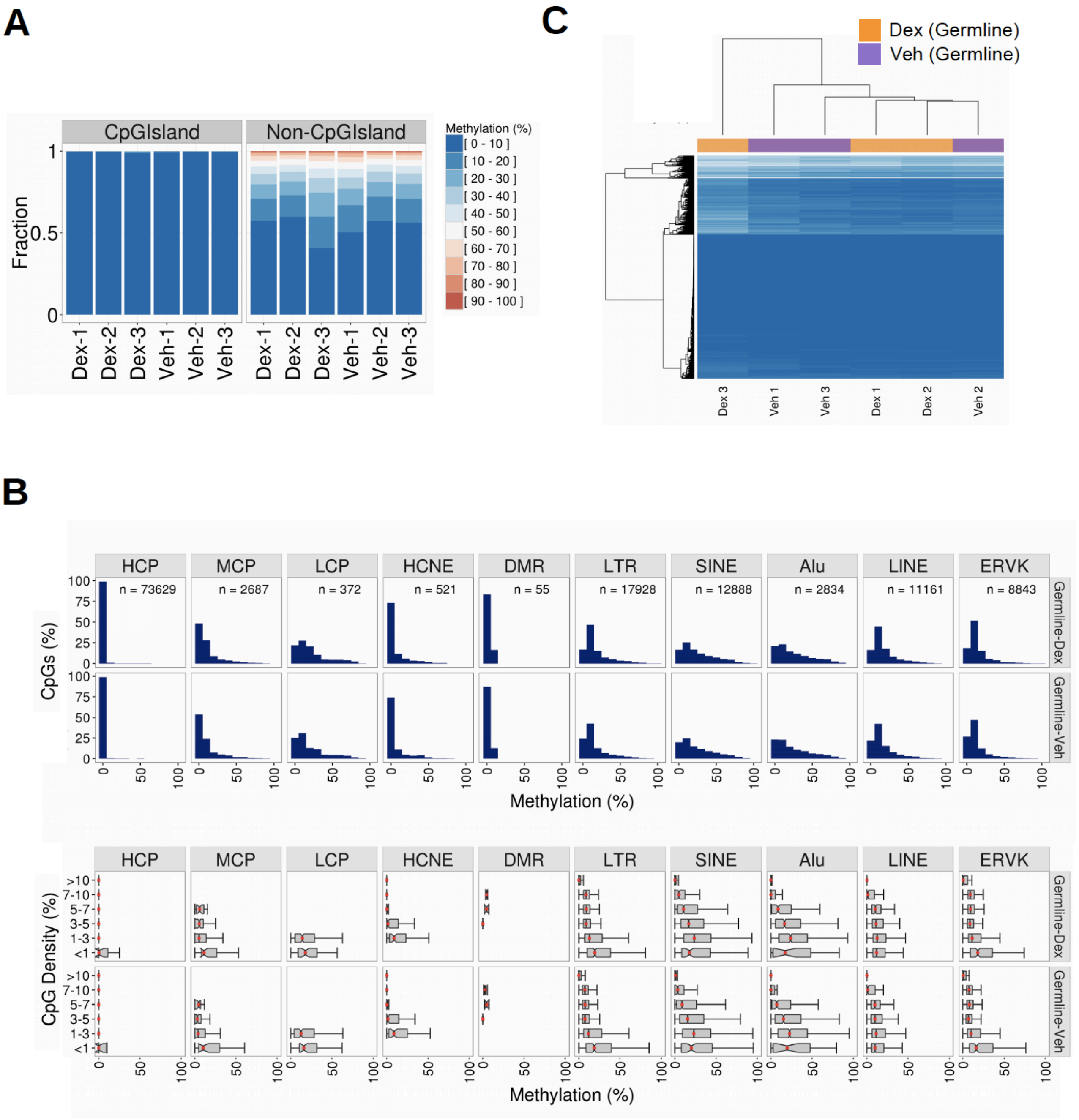
A
CpGIsland
Non-CpGIsland
Fraction
1
0.5
0
Dex-1
Dex-2
Dex-3
Veh-1
Veh-2
Veh-3
Methylation (%)
[0 - 10]
[10 - 20]
[20 - 30]
[30 - 40]
[40 - 50]
[50 - 60]
[60 - 70]
[70 - 80]
[80 - 90]
[90 - 100]
C
Dex (Germline)
Veh (Germline)
Dex 3
Veh 1
Veh 3
Dex 1
Dex 2
Veh 2
B
HCP
MCP
LCP
HCNE
DMR
LTR
SINE
Alu
LINE
ERVK
n = 73629
n = 2687
n = 372
n = 521
n = 55
n = 17928
n = 12888
n = 2834
n = 11161
n = 8843
CpGs (%)
Germline-Dex
Germline-Veh
Methylation (%)
CpG Density (%)
>10
7-10
5-7
3-5
1-3
<1
0
25
50
75
100

Figure S3: DNA methylation in the developing germline is unaffected by Dex treatment. (A) Correlation between Germline Dex replicate 1 and replicate 2 for CpG islands and all other CpGs. Equation of linear regression shown above in red. (B) Methylation (%) at three DMRs with at least 10 CpGs with ≥ 10X coverage. Median methylation is shown in bold line. Each replicate is shown as a faded line. (C) Post-hoc power test via simulation. Methylation values were modified and the power to detect these changes was calculated for 100 regions at each combination of spike-in region size and methylation change indicated on the axes. Statistical power indicated by colour with dark purple = 100%.

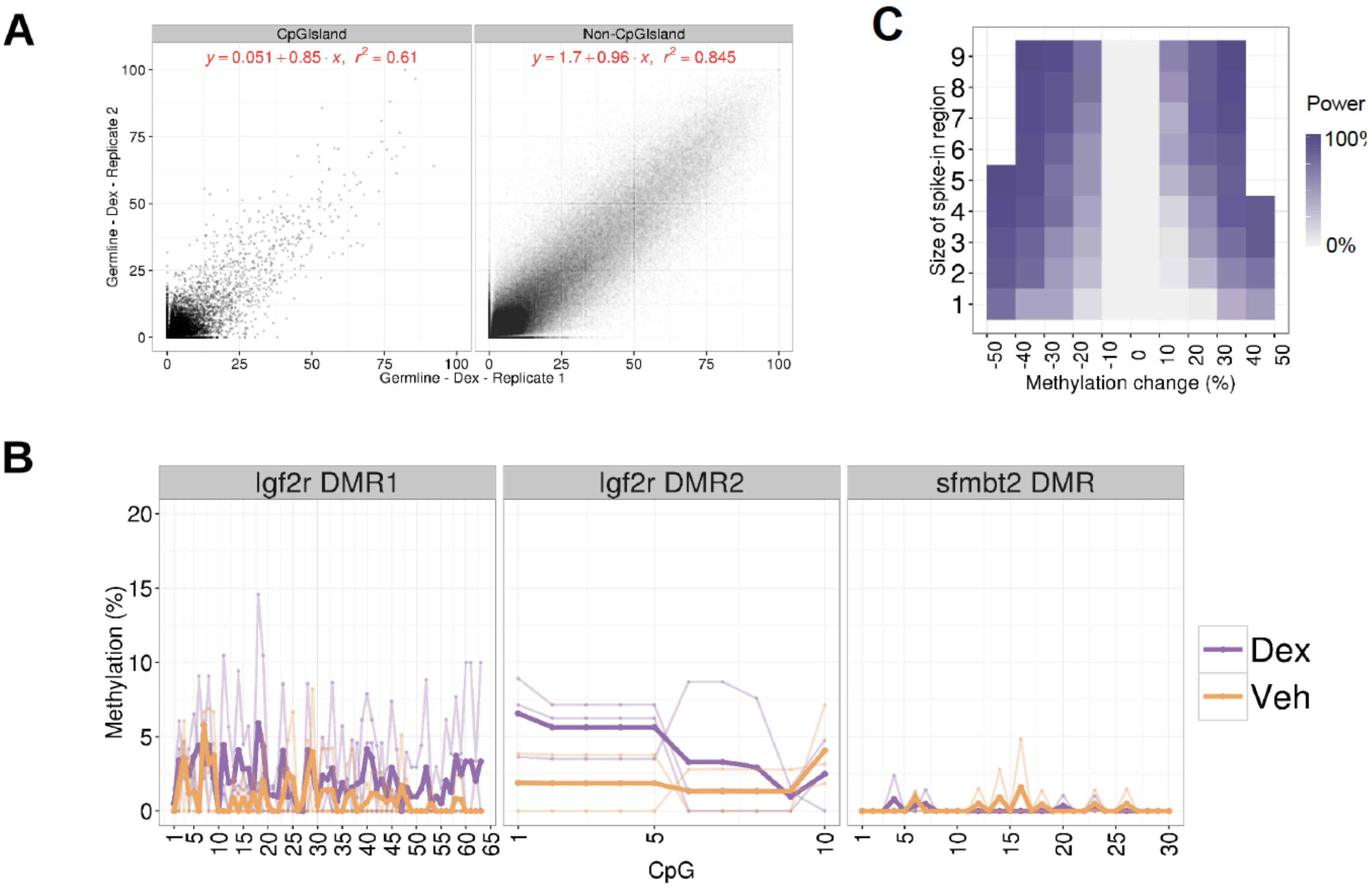

Figure S4: sRNA-Seq analysis is sufficiently powered to detect differential expression. (A) Mean expression vs. Fold change (Dex vs. Veh) for all sRNAs. (B) As per A, showing simulated *in-silico* spike-in sRNA with differences in expression between Dex and Veh - generated by random shuffling of real sRNA expression values. Colour indicates sRNA or spike-in and whether spike-in was detected as differentially expressed. (C) Estimated power to detect differentially expressed spike-in for given mean expression level and fold change.

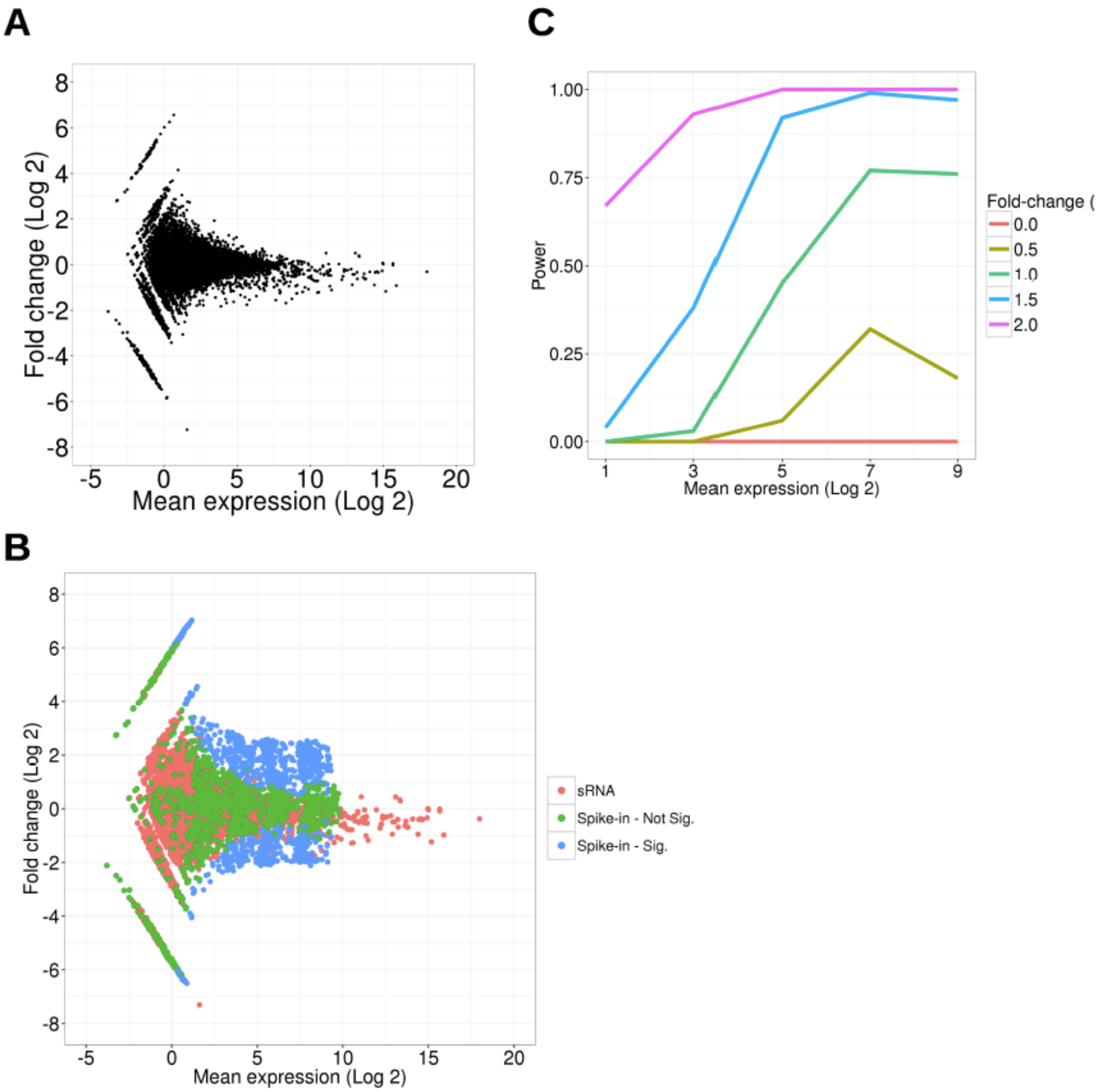
A
Fold change (Log 2)
Mean expression (Log 2)
C
Power
Mean expression (Log 2)
Fold-change (
0.0
0.5
1.0
1.5
2.0
B
Fold change (Log 2)
Mean expression (Log 2)
sRNA
Spike-in - Not Sig.
Spike-in - Sig.

Figure S5: F1-sperm sRNA expression shows consistent lack of effect of Dex treatment for two quantification methods. Hierarchical clustering of Veh and Dex samples based on miRNA, piRNA or tRNA expression indicates that samples do not cluster by treatment regardless of quantification method. Spearman's correlation Rho shown below in heatmap. (A-C) Genome alignment with BWA and quantification with featureCounts (see methods). (D-F) Quantification through iterative alignment of sRNA-Seq reads to known sRNA sequences (see methods). (A & D) miRNA. (B & E) piRNA. (C & F) tRNA.

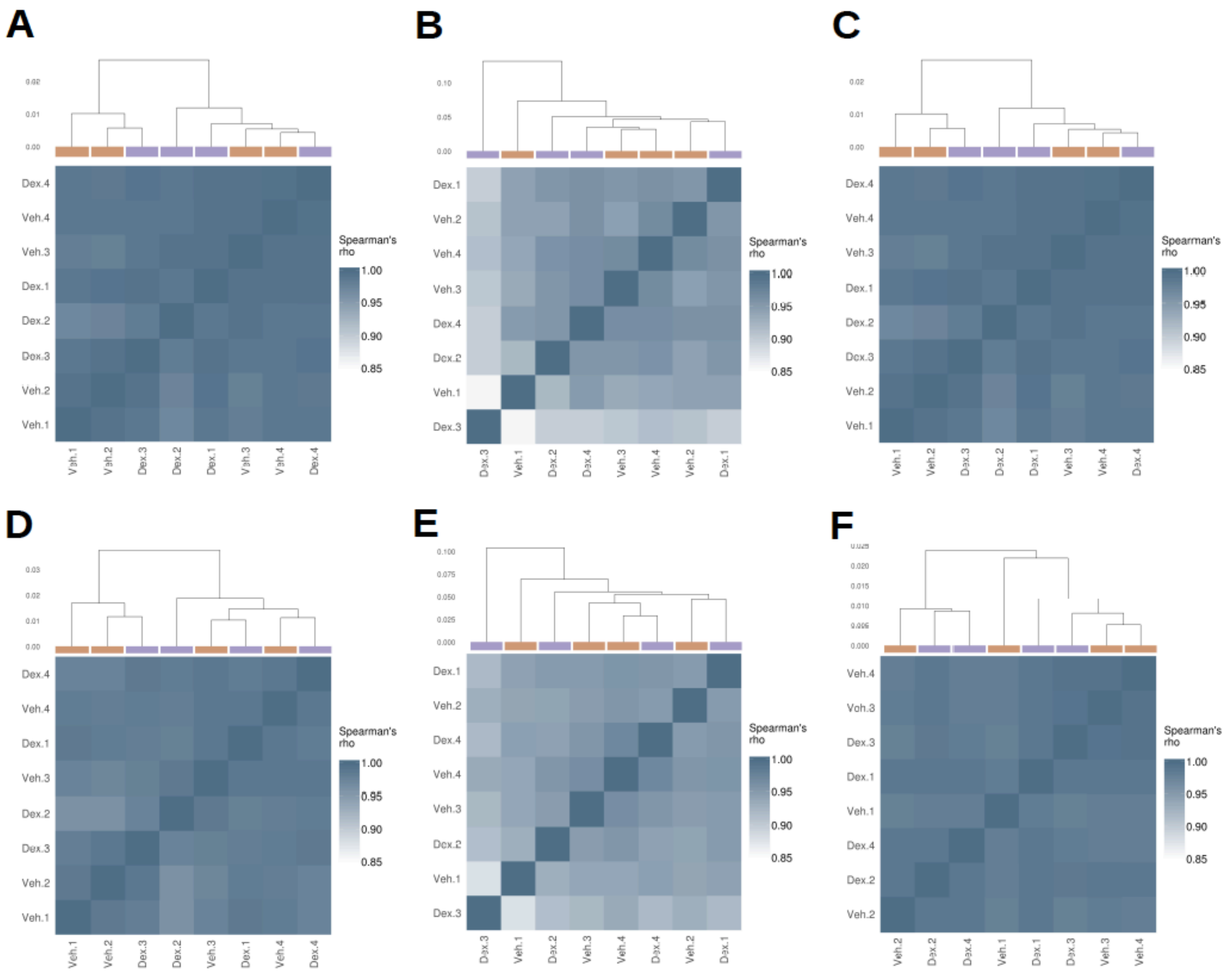

Figure S6: Fold changes between Dex and Veh sRNA expression and histone methylation and are not correlated. Scatter plots of histone methylation fold change (Dex vs. Veh) and sRNA expression fold change. Histone methylation indicated above plot. (A) miRNAs. (B). piRNAs.

A

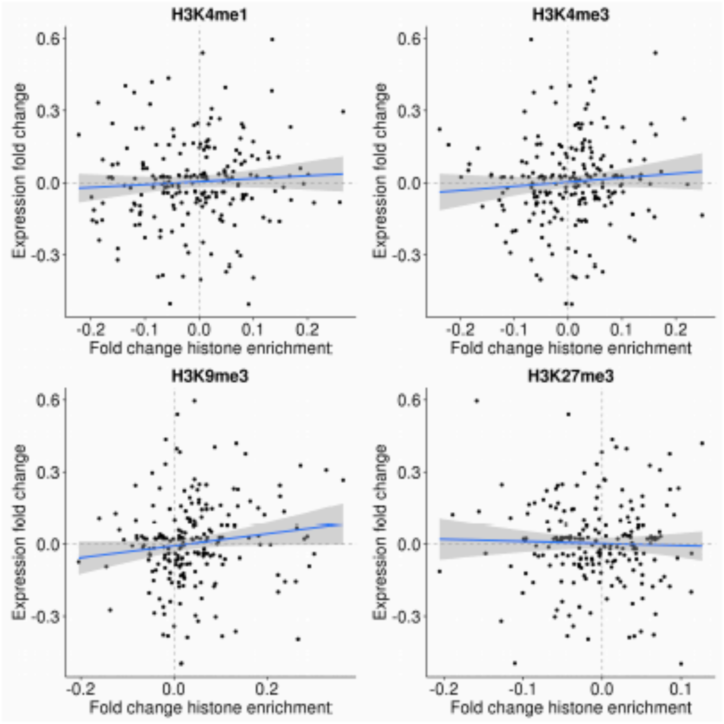

B

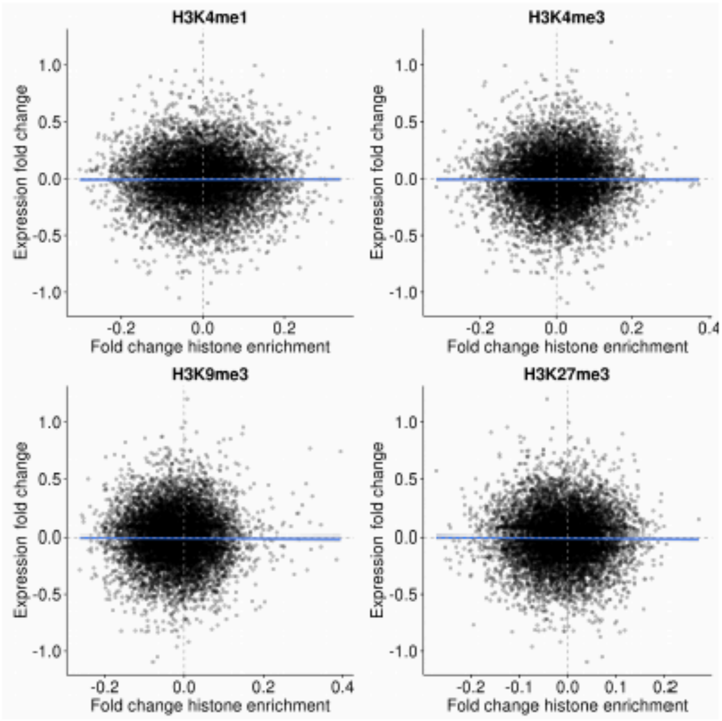

Supplement: Supplementary file 1 — Supplementary methods. ERRBS on germ cells. Figure S1. 5mC profiling in sperm. Figure S2. DNA methylation in the developing germline is unaffected by Dex treatment across the genome. Figure S3. DNA methylation in the developing germline is unaffected by Dex treatment: reproducibility and power calculations. Figure S4. sRNA-Seq analysis is sufficiently powered to detect differential expression. Figure S5. F1-sperm sRNA expression shows consistent lack of affect for Dex treatment for two quantification methods. Figure S6. Fold changes between Dex and Veh sRNA expression and histone methylation and are not correlated. (PDF 1566 kb) [file 13059_2018_1422_MOESM1_ESM.pdf]
